# Gaussian Quantum Bat Algorithm with Direction of Mean Best Position for Numerical Function Optimization

**DOI:** 10.1155/2019/5652340

**Published:** 2019-11-16

**Authors:** Xingwang Huang, Chaopeng Li, Yunming Pu, Bingyan He

**Affiliations:** ^1^Computer Engineering College, Jimei University, 185 Yinjiang Rd., Jimei District, Xiamen 361021, China; ^2^Information Engineering College, Jimei University, 185 Yinjiang Rd., Jimei District, Xiamen 361021, China

## Abstract

Quantum-behaved bat algorithm with mean best position directed (QMBA) is a novel variant of bat algorithm (BA) with good performance. However, the QMBA algorithm generates all stochastic coefficients with uniform probability distribution, which can only provide a relatively small search range, so it still faces a certain degree of premature convergence. In order to help bats escape from the local optimum, this article proposes a novel Gaussian quantum bat algorithm with mean best position directed (GQMBA), which applies Gaussian probability distribution to generate random number sequences. Applying Gaussian distribution instead of uniform distribution to generate random coefficients in GQMBA is an effective technique to promote the performance in avoiding premature convergence. In this article, the combination of QMBA and Gaussian probability distribution is applied to solve the numerical function optimization problem. Nineteen benchmark functions are employed and compared with other algorithms to evaluate the accuracy and performance of GQMBA. The experimental results show that, in most cases, the proposed GQMBA algorithm can provide better search performance.

## 1. Introduction

Recently, optimization problems are usually encountered in a mount of real-word areas such as artificial intelligence, computer science, pattern recognition, information theory, etc. Many of the actual optimization problems are frequently NP-hard problems, and searching for optimal solutions is pretty hard. It stands for the reason that it takes too long to solve these NP-hard optimization problems with traditional optimization methods. Therefore, different optimization techniques, especially bioinspired metaheuristic optimization algorithms or swarm intelligence (SI) optimization algorithms, have raised many researchers' growing interest in the past twenty years and they have proposed or developed various optimization algorithms such as particle swarm optimization (PSO) [1, 2], gravitational search algorithm (GSA) [3], ant colony optimization (ACO) [4], cuckoo search (CS) [5], and bat algorithm (BA) [6]. These algorithms are verified that they are very suited for optimizing problems such as feature selection [7, 8], task scheduling [9], unit commitment [10], artificial neural networks [11], fuzzy control [12], parameter selection and optimization [13, 14], and numerical function optimization [15]. They have also been applied to multiobjective optimization problems [16]. Compared with traditional optimization techniques, these algorithms can provide better generalization ability and parallelism. Hence, in many high-dimensional optimization problems, these algorithms outperform the traditional optimization techniques.

The bat algorithm is a relatively new nature-inspired swarm-based optimization algorithm that was proposed by Yang in 2010 [6]. This algorithm mimics the foraging behavior of bats to search the optima and it has successfully combined the merits of many well-known algorithms in a structured way, such as PSO, simulated annealing (SA) [17] and genetic algorithm (GA) [18]. BA also inherits the simplicity of PSO and it has been proved to be more efficient than its predecessor PSO and GA, especially in low-dimensional cases. Also, it is easy to implement BO in various computer languages. Hence, it has been applied to many engineering optimization problems [19]. However, due to its low population diversification, it may get trapped in local optima and premature convergence when solving high-dimensional optimization problems [20]. Therefore, to issue this deficiency, many bat algorithm variants are proposed to improve BA performance, such as CLBA [21], DLBA [22], IBA [23], HSBA [24], etc. QMBA [15] is also a variant of BA, which introduced the quantum behavior to improve the population diversity. Also, with the direction of mean best position in the later phase of searching, QMBA can convergence more quickly. QMBA is verified to be superior to the original BA and other four BA variants, including IBA, HSBA, MBA [25], and CBA [26].

However, all stochastic coefficients in the QMBA algorithm are generated using the uniform probability distribution, which can only provide a relatively small search range. Therefore, QMBA still faces a certain degree of premature convergence. To solve this issue, this article presents a technique of quantum-behaved BA directed by mean best position (QMBA) based on Gaussian distribution (GQMBA) for numerical function optimization. The employment of random Gaussian generation instead of random uniform generation in QMBA is an effective method to enhance the performance of QMBA in avoiding local solutions. In order to verify the performance of the Gaussian QMBA (GQMBA) technique, nineteen classical benchmark functions were employed, and the experimental results obtained by GQMBA over 30 trials were compared with some other algorithms mentioned in the literature.

The remainder of this article is structured as follows: the original bat algorithm is described in Sections 2 and 3 gives an overview to the QMBA.  Section 4 presents the Gaussian quantum bat algorithm with the direction of mean best position. The following Section 5 provides the simulation results and comparison of this proposed techinique. Finally, conclusions are made, and future research direction is presented in Section 6.

## 2. The Original BA Algorithm

Bat algorithm is a nature-inspired optimization algorithm inspired by foraging behavior of bats [6]. This technique is simple and easy to implement and efficient, which is swarm-based on a stochastic optimization method. When bats foraging, they search for prey and avoid obstacles by using the echolocation technique. The original BA employs a frequency-tuning approach to increase the diversification of the swarm, while, at the same time, it adopts the automatic zooming method to try to keep the balance of global search and local search during the search procedure by simulating the variations of pulse loudness and emission rates of bats when foraging. Based on three idealized rules [6], the foraging behavior of bats can be transformed to the bat algorithm. The following paragraphs present the details of BA.

In the original BA, each bat flies toward the prey, that is, moving toward the current global best position. The frequency vector (*f*_*i*_), velocity vector (*v*_*i*_), and position vector (*x*_*i*_) of the artificial bats are updated during the process of iteration using the following equations:

(1) $$\begin{array}{c} f_{i}=f_{\min}+\left(f_{\max}-f_{\min}\right)\beta , \end{array}$$

(2) $$\begin{array}{c} v_{i}^{t}=v_{i}^{t-1}+\left(x_{i}^{t}-{\text{gb}}^{t}\right)f_{i}, \end{array}$$

(3) $$\begin{array}{c} x_{i}^{t}=x_{i}^{t-1}+v_{i}^{t}, \end{array}$$

where *β* is a uniform random number in the range of [0, 1], *f*_min_ and *f*_max_ indicate the minimum and maximum frequency, respectively, gb^*t*^ means the current global best solution. With these equations, the global search capacity of BA can be guaranteed.

For the local search, to produce new solution for each bat when a solution is chosen from the current best solutions, a local random walk strategy is employed. This strategy can be described as follows:

(4) $$\begin{array}{c} x_{\text{new}}=x_{\text{old}}+\varepsilon {\bar{A}}^{t}, \end{array}$$

where *ε* is a uniform random number in the range of [−1, 1] and decides the direction of new solution. Here, *A*^*t*^ is the average loudness value of all bats at the *t*th iteration.

During the foraging process, bats will gradually adapt the values of loudness and pulse emission rate for the purpose of locating the prey. The loudness value *A*_*i*_ and pulse emission rate *r*_*i*_ can be updated in each cycle as follows:

(5) $$\begin{array}{c} A_{i}^{t+1}=\alpha A_{i}^{t}, \end{array}$$

(6) $$\begin{array}{c} r_{i}^{t+1}=r_{i}^{0}\left[1-\exp\left(-\gamma t\right)\right], \end{array}$$

where *r*^0^ indicates the initial rate of pulse emission of *i*th artificial bat. *α* and *γ* are constants. The range of *α* is [0, 1] and *γ* is a positive number (*γ* > 0). Actually, as the cooling coefficient in the SA, *α* decides the convergence of BA. For simplicity, *α*=*γ* is usually adopted in the researches.

The basic procedure of BA is described as the pseudocode illustrated in Algorithm 1.

## 3. The QMBA Algorithm

The original bat algorithm has the characteristics of simplicity, easy to implement and quick convergence; hence, it has been applied to many optimization problems. However, BA performs bad in the multimodal cases, due to its low population diversity. Through the analysis of the trajectory of artificial bats, Zhu et al. [15] proposed the quantum-behaved bat algorithm with mean best position directed. In QMBA, the quantum-behaved mutation operator can increase the diversity of swam and it also can help to avoid premature convergence. Additionally, the mean beast solution used in the later phase can quick up the convergence speed of the algorithm. The following paragraphs describe the details of QMBA [15].

QMBA is basically constructed on the basis of the original BA. The decreasing coefficient *A* and increasing coefficient *r* control the global search and local search, respectively. But the method to generate new candidate solutions is different from the original BA. The new method is described as follows:

(7) $$\begin{array}{c} x_{id}^{t}=\left\{\begin{array}{cc} x_{id}^{t-1}+\left({\text{gb}}_{d}-x_{id}^{t-1}\right)\times \eta , & \delta_{d}>\text{TH,}\\ x_{id}^{t-1}+\epsilon , & \delta_{d}\leq \text{TH,} \end{array}\right. \end{array}$$

where *η* indicates a random number uniformly distributed in the range [0, 1].

(8) $$\begin{array}{c} \delta_{d}=\left|{\text{gb}}_{d}-x_{id}^{t-1}\right|, \end{array}$$

and it represents the distance between the *d*th dimension of current global best position gb in the swarm and the position of *d*th dimension of *i*th bat, rand is a uniform random number in [0, 1]. If the distance *δ*_*d*_ is smaller than the threshold TH, the *i*th bat can fly randomly. However, if the distance *δ*_*d*_ is larger than the threshold TH, then the *i*th bat flies toward to the current global best position.

For the local search, the random walk strategy is not employed again. According to certain mutation probability *p*_*m*_, some of the bats will be mutated with quantum-behaved operator, which can be described as follows:

(9) $$\begin{array}{c} x_{id}^{t}=\left\{\begin{array}{cc} {\text{gb}}_{d}^{t}+\mu \times \left|{\text{mbest}}_{d}-x_{id}^{t}\right|\times \;\ln\left(\frac{1}{U}\right), & \text{rand}<0.5,\\ {\text{gb}}_{d}^{t}-\mu \times \left|{\text{mbest}}_{d}-x_{id}^{t}\right|\times \;\ln\left(\frac{1}{U}\right), & \text{rand}\geq 0.5, \end{array}\right. \end{array}$$

where *U* is a random number in the range [0, 1] generated by the uniform distribution and *μ* is a self-adaptive linear decreasing coefficient defined as

(10) $$\begin{array}{c} \mu =\mu_{\max}-\frac{\left(\mu_{\max}-\mu_{\min}\right)}{t_{\max}}\times t, \end{array}$$

and *μ*_max_ and *μ*_min_ are the initial and final values of *μ*. In the QMBA, *μ*_max_=1 and *μ*_min_=0.5 are adopted.

(11) $$\begin{array}{c} \text{mbest}=\frac{1}{M}\left(\overset{M}{\underset{i=1}{\sum }}P_{i1}^{t},\overset{M}{\underset{i=1}{\sum }}P_{i2}^{t},\ldots ,\overset{M}{\underset{i=1}{\sum }}P_{iD}^{t}\right), \end{array}$$

where the mbest denotes the mean best position, that is, the average value of *P*_*i*_^*t*^ positions of all artificial bats. *P*_*i*_^*t*^ represents the present best position of the *i*th bat, *M* indicates the size of swarm, and *D* represents the dimension of problem.

If a bat does not mutate with quantum-behaved operation mentioned above during the local search, then the position of the bat is updated as follows:

(12) $$\begin{array}{c} x_{i}^{t}=x_{i}^{t-1}+\left(\text{mbest}-x_{i}^{t-1}\right)\phi , \end{array}$$

where *ϕ* also denotes a random number of uniform distribution in the range [0, 1].

The pseudocode of QMBA is presented in Algorithm 2.

## 4. The GQMBA Algorithm

Various novel variants of BA have been developed to improve the performance of the original BA in recent years. Most of these BA variants generate random numbers with uniform probability distribution. However, some researches have proved that other probability distributions, such as Gaussian (normal) probability distribution, can be a good choice to improve the performance of heuristic algorithms [27–29]. In fact, any long-tail distribution helps increase the step size and distance of the random walk.

In this section, following the same direction of research, we give out a combination of QMBA and Gaussian probability distribution, which is called Gaussian QMBA (GQMBA).

A random generation that the Gaussian probability distribution with a mean of 0 and a standard deviation of 1 is utilized for initializing stochastic coefficients of GQMBA. GQMBA offers a good trade-off between the probability of having numerous small amplitudes near the present position and the small probability of having a higher amplitude. This random generation allows bats fly away from the present position and jump out of local optima. It can not only promote the accuracy of the solutions but also improve the robustness of the optimization technique. As described in Section 3, there are three uniformly distributed random sequences in the search process of QMBA. Therefore, with the application of Gaussian random generation, the GQMBA algorithm can provide a wider search space and the performance of QMBA may be improved.

In this article, stochastic numbers in GQMBA are generated using the absolute value of Gaussian probability distributions with a mean of zero and a standard deviation of one, that is, abs(*N*(0,1)) or |rand*n*|. The one-dimensional probability density function of abs(*N*(0,1)) is defined by:

(13) $$\begin{array}{c} q\left(x\right)=\frac{2}{\sqrt{2\pi }}\exp\left(-\frac{x^{2}}{2}\right),\;x\geq 0. \end{array}$$

The combination of QMBA and Gaussian probability distribution is simple but effective. Only three equations need to be modified. The three major highlights are described below.

Firstly, parameter *η* of equation (7) is modified according to the following equation:

(14) $$\begin{array}{c} x_{id}^{t}=\left\{\begin{array}{cc} x_{id}^{t-1}+\left({\text{gb}}_{d}-x_{id}^{t-1}\right)\times G, & \delta_{d}>\text{TH,}\\ x_{id}^{t-1}+\epsilon , & \delta_{d}\leq \text{TH,} \end{array}\right. \end{array}$$

where *G*=abs(*N*(0,1)).

Secondly, *U* of equation (9) is also replaced with the absolute value of the Gaussian probability distribution with a mean of zero and unit standard deviation. This quantum-behaved mutation operator now is updated according to

(15) $$\begin{array}{c} x_{id}^{t}=\left\{\begin{array}{cc} {\text{gb}}_{d}^{t}+\mu \times \left|{\text{mbest}}_{d}-x_{id}^{t}\right|\times \;\ln\left(\frac{1}{G}\right), & \text{rand}<0.5,\\ {\text{gb}}_{d}^{t}-\mu \times \left|{\text{mbest}}_{d}-x_{id}^{t}\right|\times \;\ln\left(\frac{1}{G}\right), & \text{rand}\geq 0.5, \end{array}\right. \end{array}$$

where *G*=abs(*N*(0,1)). Note that, according to equation (13), *q*(0)=0; therefore, abs(*N*(0,1)) satisfies the domain requirement of logarithmic function (>0).

Thirdly, the random number *ϕ* in equation (12) is modified according to the following equation:

(16) $$\begin{array}{c} x_{i}^{t}=x_{i}^{t-1}+\left(\text{mbest}-x_{i}^{t-1}\right)G, \end{array}$$

where *G*=abs(*N*(0,1)).

Overall, in GQMBA, the present global best solution guides the exploration phase to guarantee convergence, while the Gaussian quantum-behaved mutation operator and mean best position contribute to the exploitation phase to escape from local optimum and prevent premature convergence.

Based on the above description, the pseudocode of the GQMBA algorithm is summarized in Algorithm 3.

## 5. Experiments and Discussion

In this section, nineteen classical benchmark functions are illustrated in Tables 1–3, which are adopted to test the performance of the GQMBA algorithm. These benchmark functions are usually employed in numerical optimization techniques [15, 30–32]. In this article, the nineteen benchmark functions are grouped into three categories. The first category includes seven unimodal functions, which have only one optimal solution and are efficient to verify metaheuristic optimization techniques in terms of convergence speed and exploitation capability. The second category includes six multimodal functions, which have an exponential increasing number of local minima. Therefore, these multimodal functions are suitable for examining the local solutions avoidance and exploration capability of algorithms. The third category includes six composite functions, which are very complex with the combination of different rotated, shifted, and biased multimodal test functions. These composite functions are highly similar to the actual applications and suitable to benchmark the performance of methods in terms of balanced global search and local search. In these tables, *D* denotes the dimension of the solution space, Range is the boundary of the search space, and the global best value *f*_*m*_ is also given in column 4. Thirty independent tests are completed for every benchmark function. All the tests illustrated in this research are performed on a PC with Intel (R) Core (TM) i5-6500 3.20 GHz CPU and 8.0 GB RAM of memory, and the codes are implemented in Matlab 2014a.

Since metaheuristic algorithms belong to stochastic optimization methods, they need to be completed at least over 10 independent runs for producing meaningful statistical consequences. Besides the mean and standard deviation, statistical tests, such as Wilcoxon rank-sum test, should be conducted to verify the significance of the results based on every independent runs. In this article, the nonparametric Wilcoxon rank-sum tests are completed to verify whether there exists a statistical difference between the results obtained by GQMBA and the results searched by the other algorithms. A *p* value of less than 0.05 (<0.05) means that there exists statistical difference between the performances of the two algorithms, while a *p* value of greater than 0.05 (≥0.05) denotes that the performances are statistically similar.

**Figure 1:**
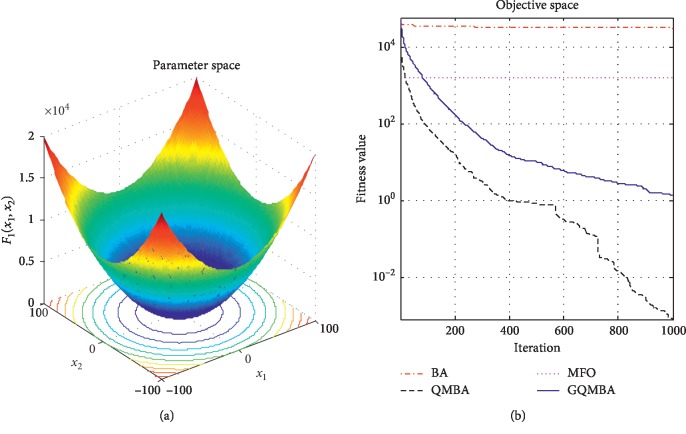
The average curve of fitness value for *F*_1_.

**Figure 2:**
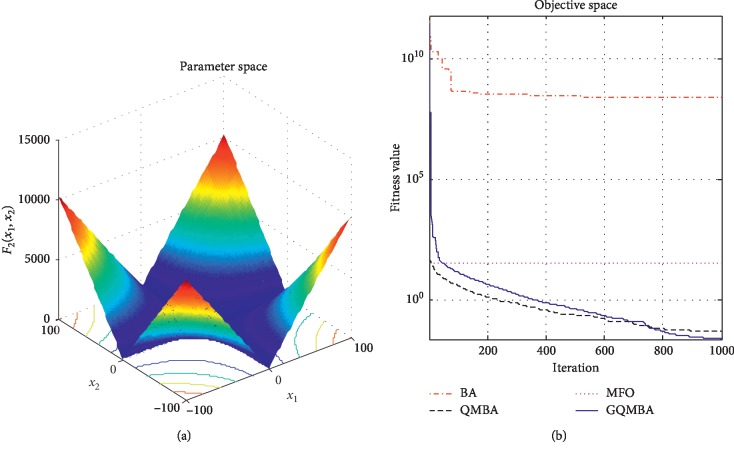
The average curve of fitness value for *F*_2_.

**Figure 3:**
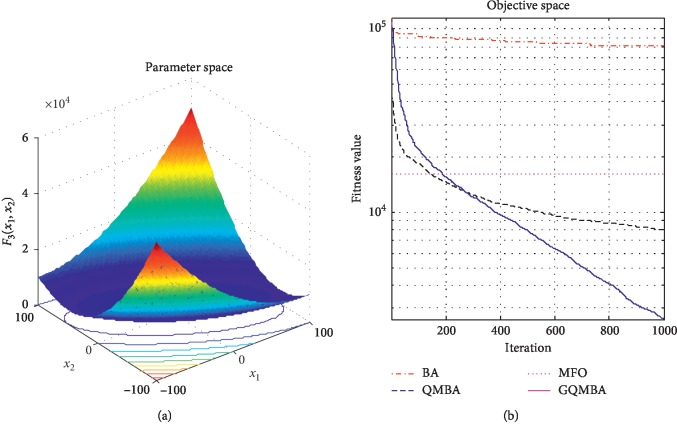
The average curve of fitness value for *F*_3_.

**Figure 4:**
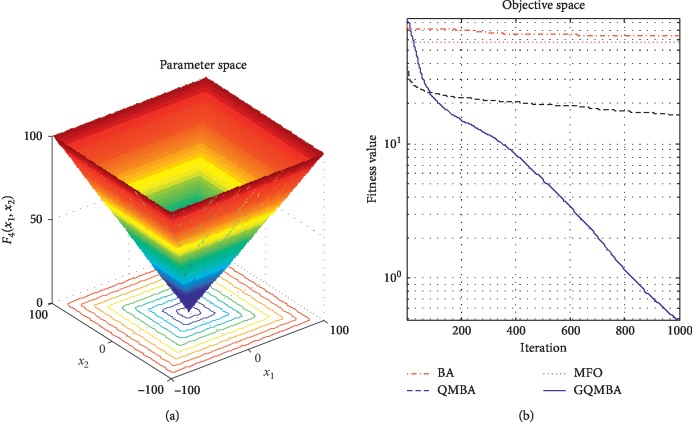
The average curve of fitness value for *F*_4_.

**Figure 5:**
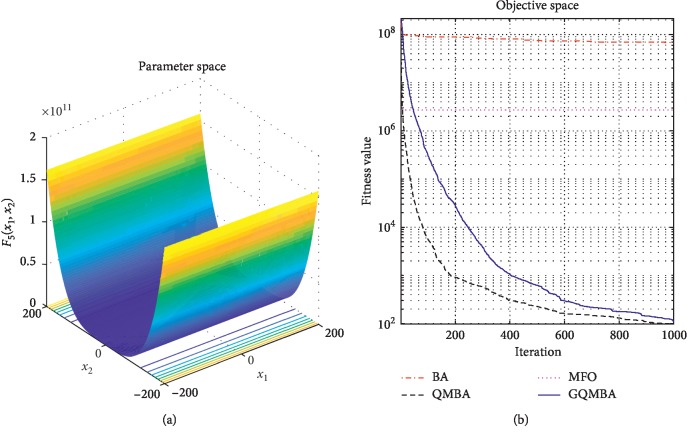
The average curve of fitness value for *F*_5_.

**Figure 6:**
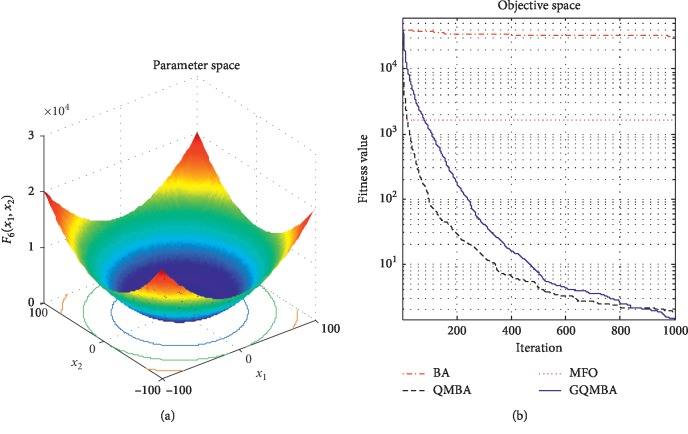
The average curve of fitness value for *F*_6_.

**Figure 7:**
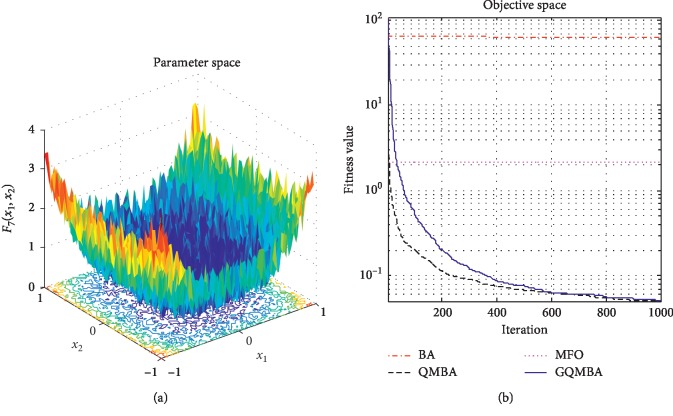
The average curve of fitness value for *F*_7_.

**Figure 8:**
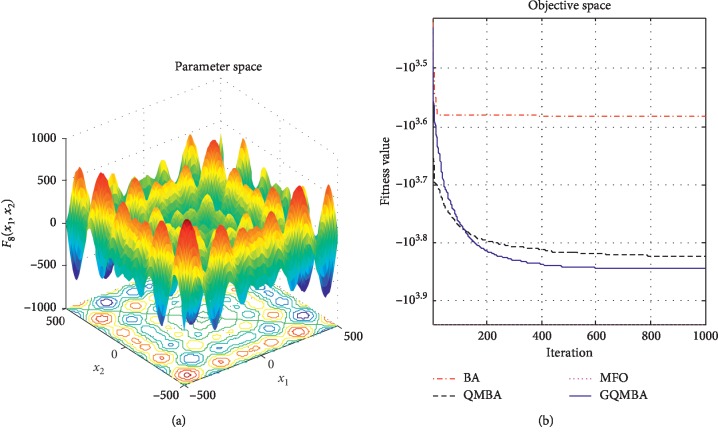
The average curve of fitness value for *F*_8_.

**Figure 9:**
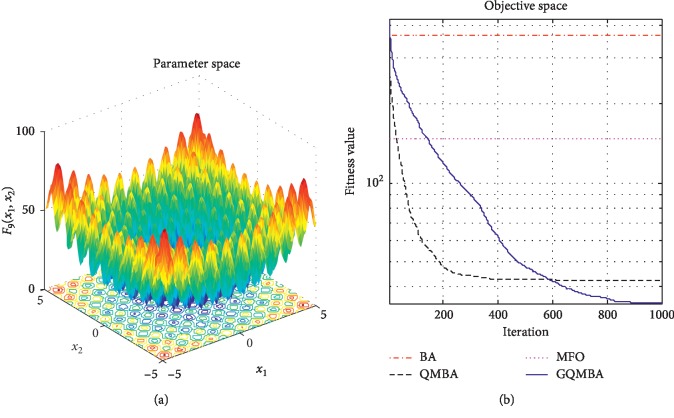
The average curve of fitness value for *F*_9_.

**Figure 10:**
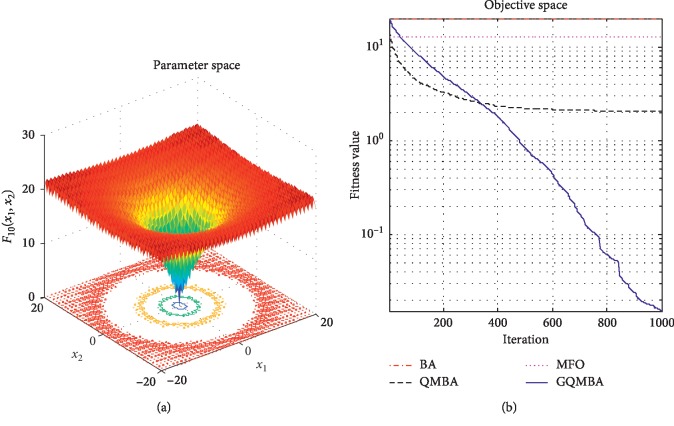
The average curve of fitness value for *F*_10_.

**Figure 11:**
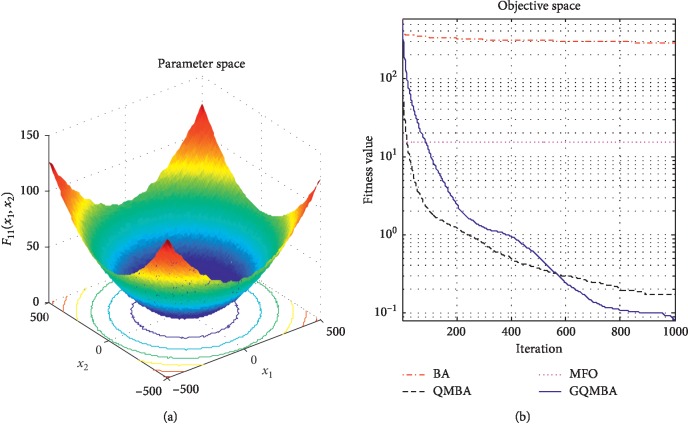
The average curve of fitness value for *F*_11_.

**Figure 12:**
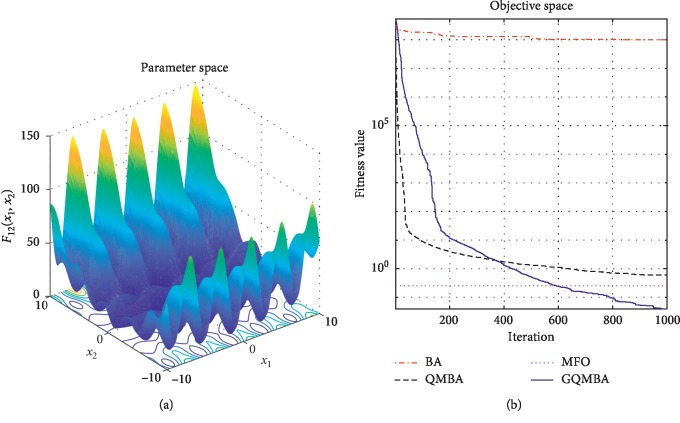
The average curve of fitness value for *F*_12_.

**Figure 13:**
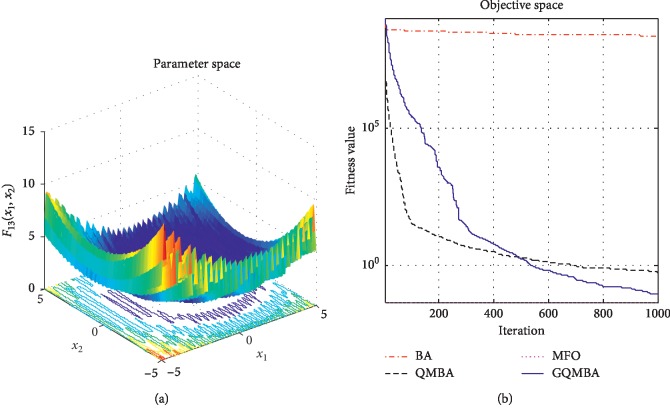
The average curve of fitness value for *F*_13_.

**Figure 14:**
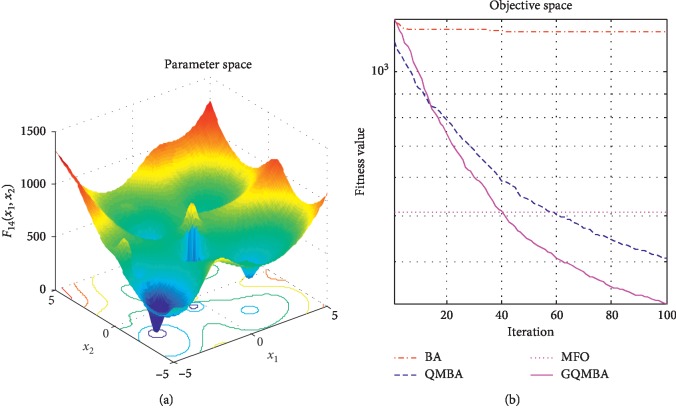
The average curve of fitness value for *F*_14_.

**Figure 15:**
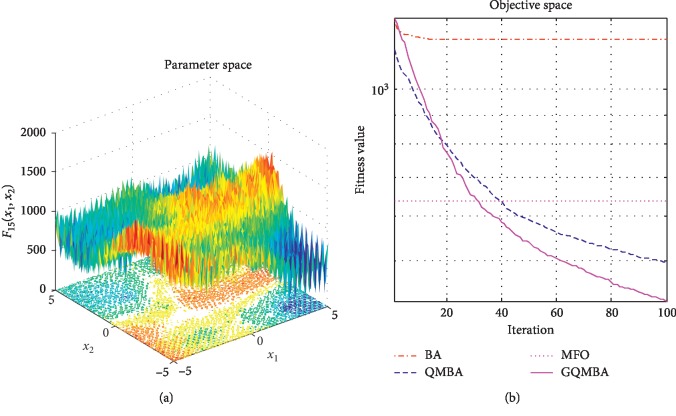
The average curve of fitness value for *F*_15_.

**Figure 16:**
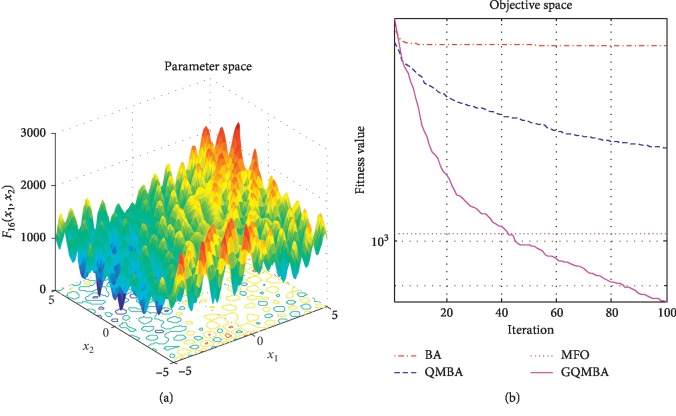
The average curve of fitness value for *F*_16_.

**Figure 17:**
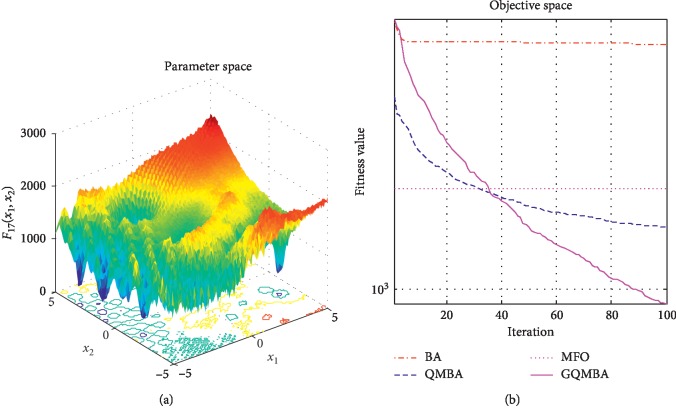
The average curve of fitness value for *F*_17_.

**Figure 18:**
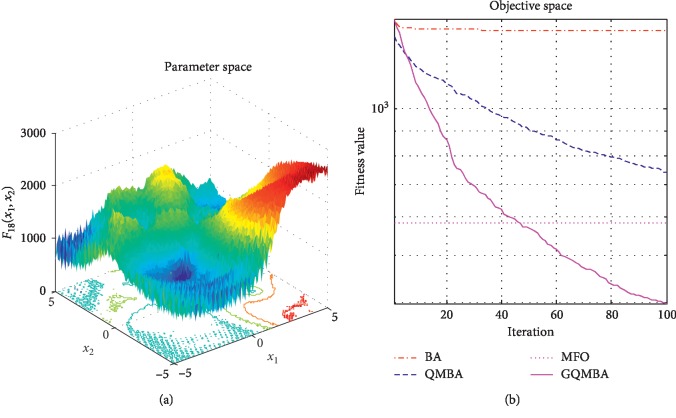
The average curve of fitness value for *F*_18_.

**Figure 19:**
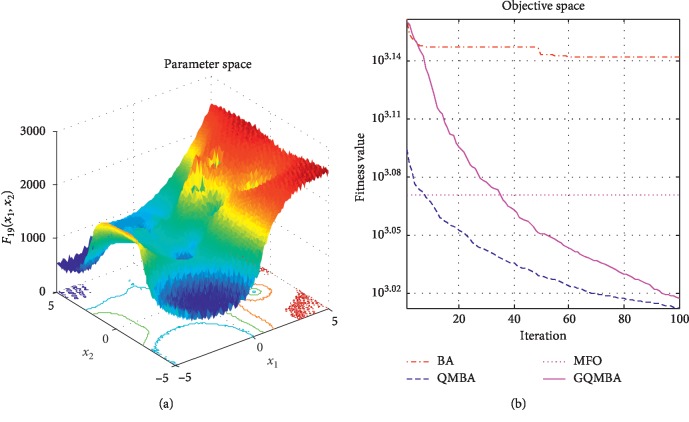
The average curve of fitness value for *F*_19_.

Considering that QMBA has been verified to be more efficient than other variants of BA [15], therefore, GQMBA is compared with the original BA and QMBA as well as a new metaheuristic algorithm MFO [30] to verify its efficiency. MFO is a novel nature-inspired heuristic algorithm. It shows high and competitive global search ability in multimodal functions and local search ability in unimodal functions. Also, MFO can balance global search and local search properly. Comparing with PSO, GSA, BA, FPA, SMS, FA, and GA, it can provide promising and competitive performance [30]. So MFO is selected as the comparative algorithm. In all swarm-based algorithms mentioned above, the maximum number of iterations is taken as 1000 for unimodal and multimodal functions, while the maximum number of iterations is taken as 100 for composite functions due to its high complexity, and the size of population is set as 50.  Table 4 shows the other parameter settings for each algorithm. As presented in Tables 1–3, nineteen classical benchmark functions are employed. The experimental results are presented in Tables 5 and 6 and Figures 1–19. Note that the optimal mean (Mean) and the optimal standard deviations (SD) of the results obtained by the four methods for each function are illustrated in bold.

As can be seen from Table 5, the GQMBA provides the best performance on most test functions, followed by the QMBA and MFO algorithms. And they are much better than the BA algorithm. To some extent, the results obtained also demonstrate that BA is easy to get trapped into local minimums when the dimension of search space is high.

The GQMBA algorithm obtains the best mean on 14 benchmark functions of 19 test functions, except *F*_1_, *F*_5_, *F*_7_, *F*_8_, and *F*_19_. At the same time, GQMBA provides the most stable solutions on fourteen benchmark functions, except *F*_1_, *F*_7_, *F*_13_, *F*_14_, and *F*_19_. The *p*-values in Table 6 illustrate that the superiority of the GQMBA algorithm is statistically significant on 9 benchmark functions, including *F*_2_, *F*_3_, *F*_4_, *F*_10_, *F*_12_, *F*_16_, *F*_17_, and *F*_18_, which cover the unimodal, multimodal, and composite benchmark functions. On the other functions, GQMBA and QMBA perform statistically similar and there is no significant difference between these two algorithms, except *F*_1_ and *F*_8_. With the analysis above, it can be concluded that the introduction of Gaussian probability distribution to QMBA is an effective mechanism and GQMBA has the significant advantage over the other three algorithms in terms of accuracy, stability, and local minimum avoidance.

Figures 1–19 demonstrate the average curves of fitness values obtained by four algorithms on nineteen functions. The values presented in these curves are the average function fitness values obtained from 30 independent tests. These figures show that the original BA and MFO converge quickly with few iterations, but they are easy to get trapped into the local optimum in many cases. These figures also demonstrate that the convergence speed of GQMBA is similar to QMBA, while GQMBA can provide better accuracy and prevent premature convergence on most benchmark functions.

In order to compare the performance in terms of computational time, the average computational time of approaches on each test function over 30 independent runs is also provided in Table 7. As can be seen from this table, for unimodal and multimodal benchmark functions, the performance of MFO in terms of computational time is best and the average computational time of GQMBA is slightly more than that of QMBA. For composite benchmark functions, the computational time of GQMBA is similar to other approaches. The obtained results comfirm that GQMBA can achieve better results with slightly more computational time.

## 6. Conclusions and Future Research Directions

In this article, novel Gaussian quantum bat algorithm with direction of mean best position (GQMBA) is proposed to simultaneously promote the search accuracy and stability of the original BA as well as QMBA. QMBA is combined with Gaussian probability distribution, which can improve the diversity, magnify the search range, and avoid falling into local optimum. Besides, GQMBA also inherits the characteristics of the original BA and QMBA, including simplicity, feasibility, and ease to implement. Nineteen benchmark functions are experimented and results show that GQMBA outperforms other algorithms. In summary, the proposed GQMBA is efficient, and it is a good alternative method to solve the numerical function optimization.

However, there still exist some deficiencies in GQMBA. One of the major deficiencies is that it has plenty of parameters to be set. Few works have already been done to reduce or make BA without any parameter as presented in [33, 34]. Therefore, in the future, we could also do some works to reduce the number of parameters in order to make GQMBA simpler but still efficient.

Finally, the proposed GQMBA algorithm might be a good choice to integrate with angle modulation method [35–37] to solve binary optimization problems. We are suggesting angle modulation method because it can change the search space from high dimension to low dimension. Hence, it can improve the accuracy and reduce the search time. In addition, the GQMBA algorithm will be applied to more optimization applications such as task scheduling, image segmentation, logic circuit design, etc.

## Figures and Tables

**Algorithm 1:**
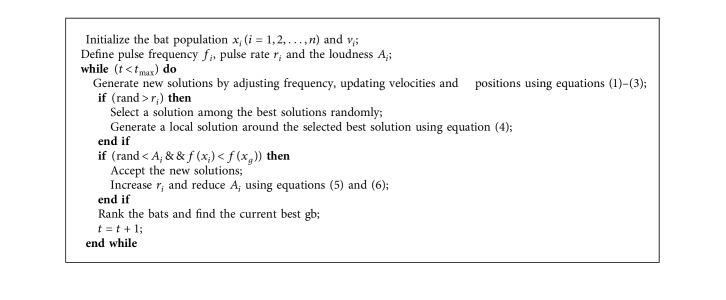
Pseudocode of the BA algorithm.

**Algorithm 2:**
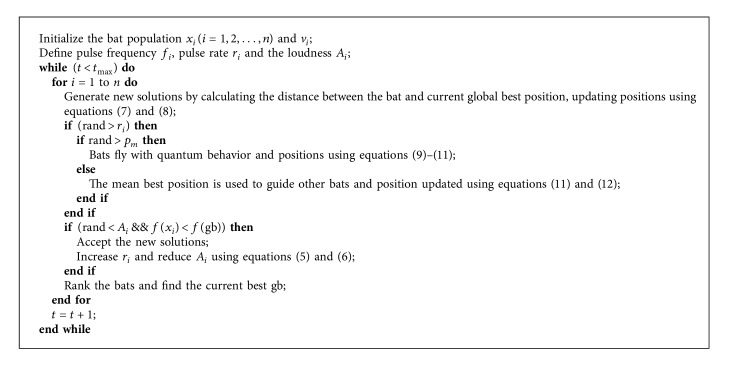
Pseudocode of the QMBA algorithm.

**Algorithm 3:**
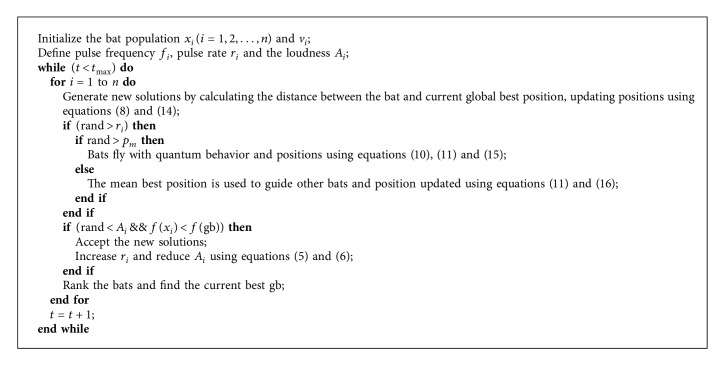
Pseudocode of the GQMBA algorithm.

**Table 1:** *F*_1_–*F*_7_ unimodal benchmark functions.

Function	*D*	Range	*f*_min_
*F*_1_(*x*)=∑_*i*=1_^*n*^*x*_*i*_^2^	30	[−100, 100]	0
*F*_2_(*x*)=∑_*i*=1_^*n*^|*x*_*i*_|+∏_*i*=1_^*n*^|*x*_*i*_|	30	[−10, 10]	0
*F*_3_(*x*)=∑_*i*=1_^*n*^(∑_*j*=1_^*i*^*x*_*j*_)^2^	30	[−100, 100]	0
*F*_4_(*x*)=max_*i*_{|*x*_*i*_|, 1 ≤ *i* ≤ *n*}	30	[−100, 100]	0
*F*_5_(*x*)=∑_*i*=1_^*n*−1^[100(*x*_*i*+1_ − *x*_*i*_^2^)^2^+(*x*_*i*_ − 1)^2^]	30	[−30, 30]	0
*F*_6_(*x*)=∑_*i*=1_^*n*^([*x*_*i*_+0.5])^2^	30	[−100, 100]	0
*F*_7_(*x*)=∑_*i*=1_^*n*^*ix*_*i*_^4^+random[0,1)	30	[−1.28, 1.28]	0

**Table 2:** *F*_8_–*F*_13_ multimodal benchmark functions.

Function	*D*	Range	*f*_min_
$F_{8}\left(x\right)=\sum_{i=1}^{n}-x_{i}\sin\left(\sqrt{\left|x_{i}\right|}\right)$	30	[−500, 500]	−418.9829 × *D*
*F*_9_(*x*)=∑_*i*=1_^*n*^[*x*_*i*_^2^ − 10 cos(2*πx*_*i*_)+10]	30	[−5.12, 5.12]	0
$F_{10}\left(x\right)=-20\;\exp\left(-0.2\sqrt{1/n\sum_{i=1}^{n}x_{i}^{2}}\right)-\exp\left(1/n\sum_{i=1}^{n}\cos\left(2\pi x_{i}\right)\right)+20+e$	30	[−32, 32]	0
$F_{11}\left(x\right)=\left(1/4000\right)\sum_{i=1}^{n}x_{i}^{2}-\prod_{i=1}^{n}\cos\left(x_{i}/\sqrt{i}\right)+1$	30	[−600, 600]	0
*F*_12_(*x*)=(*π*/*n*){10 sin(*πy*_1_)+∑_*i*=1_^*n*−1^(*y*_*i*_ − 1)^2^[1 + 10 sin^2^(*πy*_*i*+1_)]+(*y*_*n*_ − 1)^2^}+∑_*i*−1_^*n*^*u*(*x*_*i*_, 10,100,4) + ∑_*i*=1_^*n*^*u*(*x*_*i*_, 10,100,4) *y*_*i*_=1+(*x*_*i*_+1/4) $u\left(x_{i},a,k,m\right)=\left\{\begin{array}{c} k{\left(x_{i}-a\right)}^{m}x_{i}>a\\ 0-a<x_{i}<a\\ k{\left(-x_{i}-a\right)}^{m}x_{i}<-a \end{array}\right.$	30	[−50, 50]	0
*F*_13_(*x*)=0.1{sin^2^(3*πx*_1_)+∑_*i*=1_^*n*^(*x*_*i*_ − 1)^2^[1 + sin^2^(3*πx*_*i*_+1)]+(*x*_*n*_ − 1)^2^[1+ sin^2^(2*πx*_*n*_)]}+ ∑_*i*=1_^*n*^*u*(*x*_*x*_, 5,100,4)	30	[−50, 50]	0

**Table 3:** *F*_14_–*F*_19_ composite benchmark functions.

Function	*D*	Range	*f*_min_
*F*_14_(CF1)			
*f*_1_, *f*_2_, *f*_3_,…, *f*_10_=Sphere Function, [*σ*_1_, *σ*_2_, *σ*_3_,…, *σ*_10_=[1,1,1,…, 1]	30	[−5, 5]	0
[*λ*_1_, *λ*_2_, *λ*_3_,…, *λ*_10_]=[5/100, 5/100, 5/100,…, 5/100]			
*F*_15_(CF2)			
*f*_1_, *f*_2_, *f*_3_,…, *f*_10_=Griewank's Function, [*σ*_1_, *σ*_2_, *σ*_3_,…, *σ*_10_=[1,1,1,…, 1]	30	[−5, 5]	0
[*λ*_1_, *λ*_2_, *λ*_3_,…, *λ*_10_]=[5/100, 5/100, 5/100,…, 5/100]			
*F*_16_(CF3)			
*f*_1_, *f*_2_, *f*_3_,…, *f*_10_=Griewank's Function, [*σ*_1_, *σ*_2_, *σ*_3_,…, *σ*_10_=[1,1,1,…, 1]	30	[−5, 5]	0
[*λ*_1_, *λ*_2_, *λ*_3_,…, *λ*_10_]=[1,1,1,…, 1]			
*F*_17_(CF4)			
*f*_1_, *f*_2_=Ackley's Function, *f*_3_, *f*_4_=Rastrigin's Function			
*f*_5_, *f*_6_=Weierstrass Function, *f*_7_, *f*_8_=Griewank's Function	30	[−5, 5]	0
*f*_9_, *f*_10_=Sphere Function, [*σ*_1_, *σ*_2_, *σ*_3_,…, *σ*_10_=[1,1,1,…, 1]			
[*λ*_1_, *λ*_2_, *λ*_3_,…, *λ*_10_]=[5/32, 5/32, 5/32,…, 5/32]			
*F*_18_(CF5)			
*f*_1_, *f*_2_=Rastrigin's Function, *f*_3_, *f*_4_=Weierstrass Function			
*f*_5_, *f*_6_=Griewank's Function, *f*_7_, *f*_8_=Ackley's Function	30	[−5, 5]	0
*f*_9_, *f*_10_=Sphere Function, [*σ*_1_, *σ*_2_, *σ*_3_,…, *σ*_10_=[1,1,1,…, 1]			
[*λ*_1_, *λ*_2_, *λ*_3_,…, *λ*_10_]=[1/5, 1/5, 5/0.5, 5/0.5, 5/100, 5/100, 5/32, 5/32, 5/100, 5/100]			
*F*_19_(CF6)			
*f*_1_, *f*_2_=Rastrigin's Function, *f*_3_, *f*_4_=Weierstrass Function			
*f*_5_, *f*_6_=Griewank's Function, *f*_7_, *f*_8_=Ackley's Function			
*f*_9_, *f*_10_=Sphere Function	30	[−5, 5]	0
[*σ*_1_, *σ*_2_, *σ*_3_,…, *σ*_10_=[0.1, 0.2, 0.3, 0.4, 0.5, 0.6, 0.7, 0.8, 0.9, 1]			
[*λ*_1_, *λ*_2_, *λ*_3_,…, *λ*_10_]=[0.1*∗*1/5, 0.2*∗*1/5, 0.3*∗*5/0.5, 0.4*∗*5/0.5, 0.5*∗*5/100, 0.6*∗*5/100, 0.7*∗*5/32, 0.8*∗*5/32, 0.9*∗*5/100, 1*∗*5/100]			

**Table 4:** The parameter settings of BA, QMBA, MFO, and GQMBA.

Algorithms	Parameter design
BA	*f*_min_=0, *f*_max_=2, *A*=*u*(0,1), *r*=0.01, *α*=0.5, *γ*=0.5
QMBA	*A*=*u*(0,1), *r*=0.01, *α*=0.5, *γ*=0.5, TH=0.005, *P*_*m*_=0.01
MFO	Identical to the values in the original article
GQMBA	*A*=*u*(0,1), *r*=0.01, *α*=0.5, *γ*=0.5, *μ*_max_=0.9, *μ*_min_=0.5, TH=0.005, *p*_*m*_=0.01

*u*(0,1) denotes a uniform random number ranged in [0, 1].

**Table 5:** Mean and standard deviations of the benchmark functions.

*F*	BA		QMBA		MFO		GQMBA	
	Mean	SD	Mean	SD	Mean	SD	Mean	SD
*F*_1_	3.25*e* + 04	9.53*e* + 03	**8.13e − 04**	**2.87e** − **03**	1.67*e* + 03	4.61*e* + 03	1.37*e* + 00	4.07*e* + 00
*F*_2_	2.64*e* + 08	1.36*e* + 09	4.70*e* − 02	2.06*e* − 01	3.27*e* + 01	2.27*e* + 01	**2.39e − 02**	**3.77e − 02**
*F*_3_	8.15*e* + 04	3.28*e* + 04	7.99*e* + 03	4.97*e* + 03	1.61*e* + 04	8.92*e* + 03	**2.62e + 03**	**1.43e + 03**
*F*_4_	6.45*e* + 01	9.17*e* + 00	1.65*e* + 01	5.47*e* + 00	5.77*e* + 01	1.38*e* + 01	**4.81e − 01**	**7.13e − 01**
*F*_5_	6.67*e* + 07	3.21*e* + 07	**9.72e** + **01**	1.31*e* + 02	2.69*e* + 06	1.46*e* + 07	1.09*e* + 02	**1.30e + 02**
*F*_6_	3.14*e* + 04	8.06*e* + 03	1.85*e* + 00	5.70*e* + 00	1.66*e* + 03	3.78*e* + 03	**1.43e + 00**	**3.63e + 00**
*F*_7_	6.18*e* + 01	3.69*e* + 01	**5.07e − 02**	**2.41e − 02**	2.12*e* + 00	5.01*e* + 00	5.14*e* − 02	4.52*e* − 02
*F*_8_	−3.82*e* + 03	1.41*e* + 03	−6.66*e* + 03	1.61*e* + 03	**−8.70e** **+** **03**	8.74*e* + 02	−6.99*e* + 03	**7.49e + 02**
*F*_9_	3.64*e* + 02	3.34*e* + 01	4.25*e* + 01	1.68*e* + 01	1.47*e* + 02	3.23*e* + 01	**3.47e + 01**	**1.19e + 01**
*F*_10_	1.99*e* + 01	1.78*e* − 01	2.06*e* + 00	1.29*e* + 00	1.30*e* + 01	9.12*e* + 00	**1.53e − 02**	**4.69e − 02**
*F*_11_	2.83*e* + 02	8.03*e* + 01	1.71*e* − 01	3.45*e* − 01	1.51*e* + 01	4.17*e* + 01	**8.08e − 02**	**2.31e − 01**
*F*_12_	1.01*e* + 08	8.36*e* + 07	5.93*e* − 01	8.74*e* − 01	2.51*e* − 01	3.44*e* − 01	**3.88e − 02**	**8.50e − 02**
*F*_13_	2.38*e* + 08	1.46*e* + 08	5.46*e* − 01	1.56*e* + 00	4.49*e *− 02	**1.64e** **−** **01**	**8.59e − 02**	2.31*e* − 01
*F*_14_	1.22*e* + 03	1.12*e* + 02	4.06*e* + 02	2.59*e* + 02	5.08*e* + 02	**1.76e** **+** **02**	**3.27e + 02**	1.91*e* + 02
*F*_15_	1.22*e* + 03	1.24*e* + 02	4.98*e* + 02	1.92*e* + 02	6.35*e* + 02	2.46*e* + 02	**4.25e + 02**	**1.83e** **+** **02**
*F*_16_	1.59*e* + 03	1.66*e* + 02	1.25*e* + 03	1.96*e* + 02	1.02*e* + 03	2.02*e* + 02	**8.66e + 02**	**1.60e** **+** **02**
*F*_17_	1.44*e* + 03	1.00*e* + 02	1.10*e* + 03	7.58*e* + 01	1.16*e* + 03	1.61*e* + 02	**9.80e + 02**	**1.46e** **+** **02**
*F*_18_	1.45*e* + 03	1.29*e* + 02	7.44*e* + 02	3.69*e* + 02	5.83*e* + 02	3.46*e* + 02	**4.00e + 02**	**2.61e** **+** **02**
*F*_19_	1.39*e* + 03	8.79*e* + 01	**1.03e** + **03**	**4.32e** + **01**	1.18*e* + 03	6.28*e* + 01	1.04*e* + 03	5.40*e* + 01

**Table 6:** *p*-values of the Wilcoxon rank-sum test over all runs.

F	GQMBA	MFO	QMBA	BA
*F*_1_	0.0150	0.0176	N/A	3.0199*e* − 11
*F*_2_	N/A	5.5727*e* − 10	0.0207	3.0199*e* − 11
*F*_3_	N/A	8.8411*e* − 07	3.8053*e* − 07	3.0199*e* − 11
*F*_4_	N/A	3.0199*e* − 11	3.0199*e* − 11	3.0199*e* − 11
*F*_5_	*0.4376*	3.3679*e* − 04	N/A	3.0199*e* − 11
*F*_6_	N/A	*0.3255*	0.0133	3.0199*e* − 11
*F*_7_	*0.2707*	5.5611*e* − 04	N/A	3.0199*e* − 11
*F*_8_	7.1186*e* − 09	N/A	0.0168	4.1997*e* − 10
*F*_9_	N/A	3.0199*e* − 11	*0.1188*	3.0199*e* − 11
*F*_10_	N/A	2.3897*e* − 08	7.7725*e* − 09	1.4110*e* − 09
*F*_11_	N/A	*0.4290*	0.0251	3.0199*e* − 11
*F*_12_	N/A	3.5708*e* − 06	4.0840*e* − 05	3.0199*e* − 11
*F*_13_	*0.0850*	N/A	9.7917*e* − 05	3.0199*e* − 11
*F*_14_	N/A	5.2640*e* − 04	*0.2340*	3.0199*e* − 11
*F*_15_	N/A	0.0023	*0.1055*	3.0199*e* − 11
*F*_16_	N/A	0.0021	2.1947*e* − 08	3.6897*e* − 11
*F*_17_	N/A	4.0840*e* − 05	8.1200*e* − 04	3.0199*e* − 11
*F*_18_	N/A	0.0083	1.0407*e* − 04	3.3384*e* − 11
*F*_19_	*0.3871*	2.2273*e* − 09	N/A	4.0772*e* − 11

N/A means not applicable. *p* ≥ 0.05 have been italized.

**Table 7:** Average computational time of approaches on each benchmark function.

*f*	BA	MFO	QMBA	GQMBA
*f*_1_	1.10036	0.469359	0.889615	0.929227
*f*_2_	1.04243	0.524513	0.921171	0.980138
*f*_3_	3.0765	2.43098	2.88777	2.95636
*f*_4_	1.07779	0.567324	1.00172	1.03555
*f*_5_	1.22965	0.71997	1.14489	1.19135
*f*_6_	1.09087	0.599222	1.04263	1.08032
*f*_7_	1.35753	0.886334	1.29639	1.33972
*f*_8_	1.15973	0.654205	1.10923	1.14526
*f*_9_	1.18425	0.672098	1.11233	1.16105
*f*_10_	1.23347	0.716191	1.16477	1.19911
*f*_11_	1.29489	0.754089	1.20586	1.25417
*f*_12_	1.99625	1.43408	1.90856	1.95945
*f*_13_	1.98464	1.42408	1.89719	1.93967
*f*_14_	18.8752	16.5838	22.6735	17.1589
*f*_15_	18.325	18.08	18.72	18.7628
*f*_16_	19.9876	25.5575	23.0384	21.1629
*f*_17_	27.0739	24.9261	23.0546	28.294
*f*_18_	22.5187	22.7105	22.3053	22.7562
*f*_19_	22.4076	21.9222	22.2938	22.2832

## Data Availability

No data were used to support this study.
